# Role of cerebellar cortex in associative learning and memory in guinea pigs

**DOI:** 10.1515/biol-2022-0471

**Published:** 2022-09-14

**Authors:** Rui Li, Qi Li, Xiaolei Chu, Lan Li, Xiaoyi Li, Juan Li, Zhen Yang, Mingjing Xu, Changlu Luo, Kui Zhang

**Affiliations:** Department of Traditional Chinese Medicine, Guizhou Provincial People’s Hospital, Zhongshan East Road 83, Guiyang 550001, Guizhou, China; Department of Rehabilitation Medicine, Tianjin Hospital Tianjin University, Jiefang South Road 406, Tianjin 300211, Tianjin, China; Academy of Medical Engineering and Translational Medicine, Tianjin University, Tianjin 300072, Tianjin, China; Department of Clinical Laboratory, Guizhou Provincial People’s Hospital, Zhongshan East Road 83, Guiyang 550001, Guizhou, China; Department of Neuroelectrophysiology, Guizhou Provincial People’s Hospital, Zhongshan East Road 83, Guiyang 550001, Guizhou, China; Department of Using Quality Management, Guizhou Provincial People’s Hospital, Zhongshan East Road 83, Guiyang 550001, Guizhou, China; Department of Orthopedics, Guizhou Provincial People’s Hospital, Zhongshan East Road 83, Guiyang 550001, Guizhou, China; Department of Rehabilitation, Guizhou Provincial People’s Hospital, Zhongshan East Road 83, Guiyang 550001, Guizhou, China

**Keywords:** cerebellar cortex, delay eyeblink conditioning, lateral pontine nuclei, middle cerebellar peduncle, trace eyeblink conditioning

## Abstract

Time-related cognitive function refers to the capacity of the brain to store, extract, and process specific information. Previous studies demonstrated that the cerebellar cortex participates in advanced cognitive functions, but the role of the cerebellar cortex in cognitive functions is unclear. We established a behavioral model using classical eyeblink conditioning to study the role of the cerebellar cortex in associative learning and memory and the underlying mechanisms. We performed an investigation to determine whether eyeblink conditioning could be established by placing the stimulating electrode in the middle cerebellar peduncle. Behavior training was performed using a microcurrent pulse as a conditioned stimulus to stimulate the middle cerebellar peduncle and corneal blow as an unconditioned stimulus. After 10 consecutive days of training, a conditioned response was successfully achieved in the Delay, Trace-200-ms, and Trace-300-ms groups of guinea pigs, with acquisition rates of >60%, but the Trace-400-ms and control groups did not achieve a conditioned stimulus-related blink conditioned response. It could be a good model for studying the function of the cerebellum during the establishment of eyeblink conditioning.

## 1 Introduction

The classic eyeblink conditioning combines events associated with time cognition using conditioned stimulus and unconditioned stimulus and, therefore, is widely used as an associative learning and memory model for studying the mechanisms of temporal information processing in specific brain areas [1,2]. Delay and trace conditioning are distinguished not by the interval between stimuli, which can be identical, but by the difference in the offset of the stimuli. In trace conditioning, the onset and offset of the conditioned stimulus occur before the onset of the unconditioned stimulus, and there is a gap (or “trace”) between them. In delay conditioning, the onset between the conditioned and unconditioned stimuli is different, but the offset is usually the same, i.e., they end together, and there is an overlap between the two stimuli. The two behavioral models differ according to the presence of a time interval between stimuli. As a result, there is a gap between the onsets of the conditioned and unconditioned stimuli. The length of this gap can be used to study the ability of specific brain areas to process information related to temporal information processing.

Patients with autism spectrum disorders [3], cerebellar diseases [4], cerebellar degeneration [5], migraine [6], fetal alcohol syndrome [7], schizophrenia [8,9], severe depression [10], and other neuropsychiatric disorders [11,12,13] all show the abnormal ability of temporal information processing. Thus, using the eyeblink conditioning model to study the brain areas involved in temporal information processing would help to understand further the neurobiological mechanism of time-related learning and memory in mammalian animals [14,15].

Previous studies have suggested that temporal information processing mainly occurs in cerebral neural circuits, including the thalamus, cerebral cortex, medial prefrontal cortex, and hippocampus [16,17,18]. In addition, similar to the cerebral cortex, the cerebellum also exerts advanced functions in temporal information processing. Indeed, patients with cerebellar injury develop motor sequence learning disorders [19,20], suggesting the importance of the cerebellum in time estimation. In addition, neuroimaging studies have shown significant differences in the area of cerebellar activation before and after sequence training and learning [21,22]. Cerebellar lesions can lead to a wide range of clinical cognitive impairments, and the most common is impaired temporal information processing [23,24]. In addition, pathological changes in cerebellar disorders, including cerebellar hypoplasia [25], space-occupying intracranial lesions [26,27], and trauma [26], among others, can also lead to dysfunction or loss of temporal information processing-related abilities. Animal experiments have shown that decerebrated guinea pigs (the cerebral cortex and subcortical hippocampus were removed completely, and only the brainstem and cerebellum were left) still acquired conditioned responses [28]. Recordings of basket cell discharge have shown that the activation of certain cerebellar neurons is closely associated with classical eyeblink conditioning [29,30,31]. In addition, physical damage to the cerebellar cortex significantly inhibits the ability of rabbits to establish eyeblink conditioning [32,33,34,35,36,37,38,39]. These results all demonstrate that the cerebellum is closely related to temporal information processing, but whether it independently participates in temporal information processing in associative learning and memory is still controversial. Therefore, exploring the role of the cerebellar cortex in associative learning and memory would help understand the mechanism of specific brain areas in handling time-related events. Based on the foregoing, this issue has attracted considerable research in the field of neuroscience.

Classical eyeblink conditioning often uses peripheral sensory stimuli as the conditioned stimulus and conducts the signal via visual or auditory pathways to the thalamus, cerebral cortex, or medial prefrontal cortex. With respect to eyeblink conditioning established via the forebrain–cerebellum circuit, the conditioned stimulus is often affected by many external factors, and this limits the study of specific brain areas in related neural circuits. Since neurons or circuits can hardly be analyzed specifically, determining whether the cerebellar cortex is directly involved in associative learning and memory is therefore difficult. Neuroanatomy has shown that the middle cerebellar peduncle, with mossy fiber as its main component, is one of the major afferent fibers in the cerebellar cortex. It is constituted by the pontine-cerebellar fiber derived from the lateral pontine nucleus, and it ends at the cerebellar cortex [40].

At present, it is still unclear how mammals connect two independent events based on time and by which neurobiological mechanism they process related information and take appropriate action within the corresponding time [41,42]. The eyeblink conditioning model has been widely used in exploring the mechanism of certain brain areas in learning and memorizing [43]. As previously used by several authors and studies, we used microcurrent pulse and photosensitive receptors to substitute traditional sound and light as the conditioned stimulus to directly stimulate the middle cerebellar peduncle and used corneal blow as the unconditioned stimulus, as previously described for different parts of the brain [18,44,45,46,47,48]. This design successfully established a delay eyeblink conditioning model and trace eyeblink conditioning models within a certain time interval range. Our results suggested that the cerebellar cortex could accomplish the process of associative learning and memory independently for time-related events. A similar approach was used by Steinmetz et al. [49], who showed conditioned blinks using mossy fiber stimulation in the rabbit, and by Swain, Shinkman, and Thompson in the 1990s [50], who used electrical stimulation of the cerebellar cortex to mediate conditioned eyeblinks in the rabbit.

Thus, this study aimed to use a microcurrent pulse to directly stimulate the middle cerebellar peduncle and observe whether the cerebellar cortex could establish an eyeblink conditioning independently, and analyze the cerebellum’s role in recognizing time in Guinea pigs. Using induced action potential on mossy fiber as the conditioned stimulus, we prevented persistent neuronal activity generated from mixed information input to different forebrain structures and, therefore, avoided this influence on the establishment of eyeblink conditioning. Briefly, this study substituted traditional sound and light stimuli with electrophysiology as the conditioned stimulus. It also investigated whether the cerebellar cortex could establish eyeblink conditioning and its amplitude under such stimulation. By doing this, we further revealed the role and mechanism of the cerebellar cortex in associative learning and memory. This study provides conclusive evidence for the function of the cerebellum in advanced temporal information processing. Our study will facilitate the application of neurobiology in clinical diagnosis and treatment. It will also provide a theoretical foundation for managing cerebellar cognitive dysfunction, particularly temporal information processing. This study could help determine the impact of the location of an injury on cognitive behavior and infer whether the lesion location could impact the prognosis. Eyeblink conditioning was used in previous studies of various medical conditions [11,12,13].

## 2 Materials and methods

### 2.1 Experimental animals

The animals were provided by the Laboratory Animal Center (license #SCXK (Chongqing) 2007-0001). Male guinea pigs aged 4–5 months, weighing 400–450 g and without obvious eye diseases, were selected for electrode neuronal stimulation. All animals were kept separately in cages with dry sawdust matting and free access to food and water. Animals were reared in an environment of 20–25°C temperature, 50–80% relative humidity, and a 12/12 h light-dark cycle.


**Ethical approval:** The research related to animal use has been complied with all the relevant national regulations and institutional policies for the care and use of animals, and was approved by the Ethics Committee of Guizhou Provincial People’s Hospital (No. 2015014).

### 2.2 Intracranial implantation of the stimulating electrode

Guinea pigs were anesthetized by intraperitoneal injection of ketamine (80 mg/kg) and phenothiazine (5 mg/kg). Thirty guinea pigs were selected for implantation shocks. The guinea pigs were divided into (1) delay group, (2) Trace-200ms group, (3) Trace-300ms group, (4) Trace-400ms group, and (5) control group, with six animals per group, 30 in total. In the end, one animal in the Trace-200ms group and one in the Trace-400ms group were implanted with electrodes but their placement was incorrect. Therefore, 28 guinea pigs were included in the final behavioral groups: (1) Delay group, *n* = 6; (2) Trace-200ms group, *n* = 5; (3) Trace-300ms group, *n* = 6; (4) Trace-400ms group, *n* = 5; and (5) control group, *n* = 6. For electrode implantation, the head was fixed in a stereotactic apparatus (SR-6N, Narishige Scientific Instrument, Japan). Then, using the bregma suture as the zero-reference point, a welded stimulating electrode (A-M Systems, Sequi, USA, external diameter: 0.7 mm, coating diameter: 330.2 mm, and internal diameter: 0.254 mm) was implanted into the brain at 15 mm posterior to the bregma, 3.5 mm laterally (left) from the midline, and 7 mm ventrally from the bregma line. The electrode tip was fixed using dental cement in the left middle cerebellar peduncle. A reference electrode was also implanted in the left middle cerebellar peduncle. The guinea pigs were observed for 1 week after surgery. Those with no significant infection and high activity were taken for behavior training.

### 2.3 Behavior training

**Figure 1:**
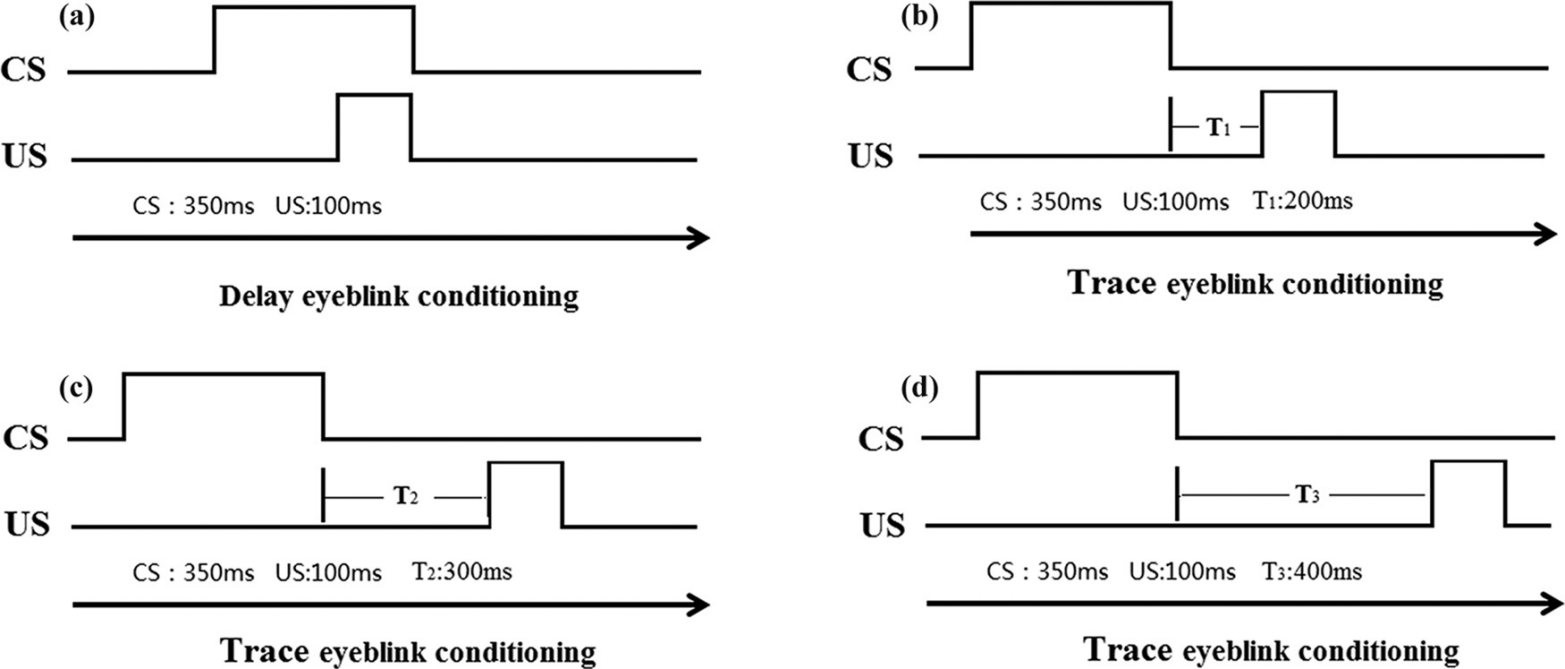
The training patterns of four traditional eyeblink conditioning groups. Based on whether the time of conditioned stimulus and unconditioned stimulus overlapped, eyeblink conditioning could be further divided into delay eyeblink conditioning and trace eyeblink conditioning. In delay eyeblink conditioning, (a) the conditioned stimulus happens prior to the unconditioned stimulus and ends with the unconditioned stimulus, whereas in trace eyeblink conditioning, (b–d) a time interval presents between the ending of the conditioned stimulus and the starting of the unconditioned stimulus.

Thirty male guinea pigs that successfully underwent the implantation were randomized (lottery method) into the experimental groups (Delay, Trace-200ms, Trace-300ms, and Trace-400ms groups) and the control group (*n* = 6/group). According to whether there was a fixed time relationship between CS and US, they were divided into the experimental and control groups. According to the time interval (trace interval, TI) between CS and US, they were divided into the (1) the Delay group, (2) Trace-200ms group, (3) Trace-300ms group, and (4) Trace-400ms group, for a total of five groups (including the control group) with six animals in each group. For adaptation, all guinea pigs were placed in a shielding cabinet with light and sound insulation for 2 days, 60 min each day, without any stimulation. After adaptation, the animals underwent 10 consecutive days of behavior training, with a session of 100 stimulations each day. A given animal was trained at the same time each day. A conditioned stimulus was produced using a microcurrent pulse generated from a YC-2 stimulator (Chengdu Instrument, China) and an isolation unit (ISO-Flex, AMPI, Israel) to stimulate the right middle cerebellar peduncle. The output intensity was min 40%, which could initiate a blink reaction (based on the preliminary experiment, this was obtained by increasing the output intensity until eyelid closing could be measured). For the electrical stimulations, the waveform was a train of 0.1 ms pulses delivered at 200 Hz for 350 ms. The waveform was a square wave, and the pulse was a 200 Hz series of single-pulse current (pulse interval time: 4.9 ms, single pulse width: 0.1 ms, and pulse: 70 groups). A 100-ms pure oxygen flow was used as the unconditioned stimulus, and the outlet pressure was strictly adjusted to 3 psi by a pressure-reducing valve. The time interval for trace eyeblink conditioning is shown in Figure 1 for the different experimental groups. For the control group, the time interval between the conditioned and unconditioned stimuli was randomly chosen within 10–40 s (20 s on average). There was no intrinsic relationship between the times of occurrence of the conditioned stimulus and unconditioned stimuli stimulus events.

### 2.4 Data collection and recording

For neuronal stimulation by the electrode, the end of a frog heart clamp with a surgical suture was connected to a muscular-tension transducer (JZ100, Beijing, China), and the head was clipped to the free edge of the left upper eyelid of the guinea pigs, with the intensity maintained at a level that would allow the guinea pig to open the eyelid naturally. The movement could initiate tensional changes that could be transduced into an electric signal to record eyelid movements. Taking conditioned stimulus onset as the zero points, the baseline signal was taken from the average signal of 1 ms within 350 ms before conditioned stimulus onset. Blinks satisfying the following two conditions were determined as effective eyeblinks: (1) the upper eyelid movement was ≥baseline +1 mV; and (2) the total time was ≥15 ms. The analytical parameters were active eyeblinks within 200 ms before unconditioned stimulus onset and the magnitude of the difference between the maximum active eyeblinks within 200 ms before unconditioned stimulus onset eyeblink signal and the conditioned stimulus onset signal. The magnitude of the difference was based on the nictitating membrane response classical pathway [39].

### 2.5 Statistical analysis

Data were input into Microsoft Excel, and statistical analysis was performed using SPSS 18.0 (IBM, Armonk, NY, USA). Data were presented as means ± standard deviations. Graphs were plotted using Excel. Data were analyzed by the *t-*test and one-way ANOVA, and statistical significance was defined as *P* < 0.05.

## 3 Results

### 3.1 Successful eyeblink conditioning modeling using microcurrent pulse stimulation of the middle cerebellar peduncle

**Figure 2:**
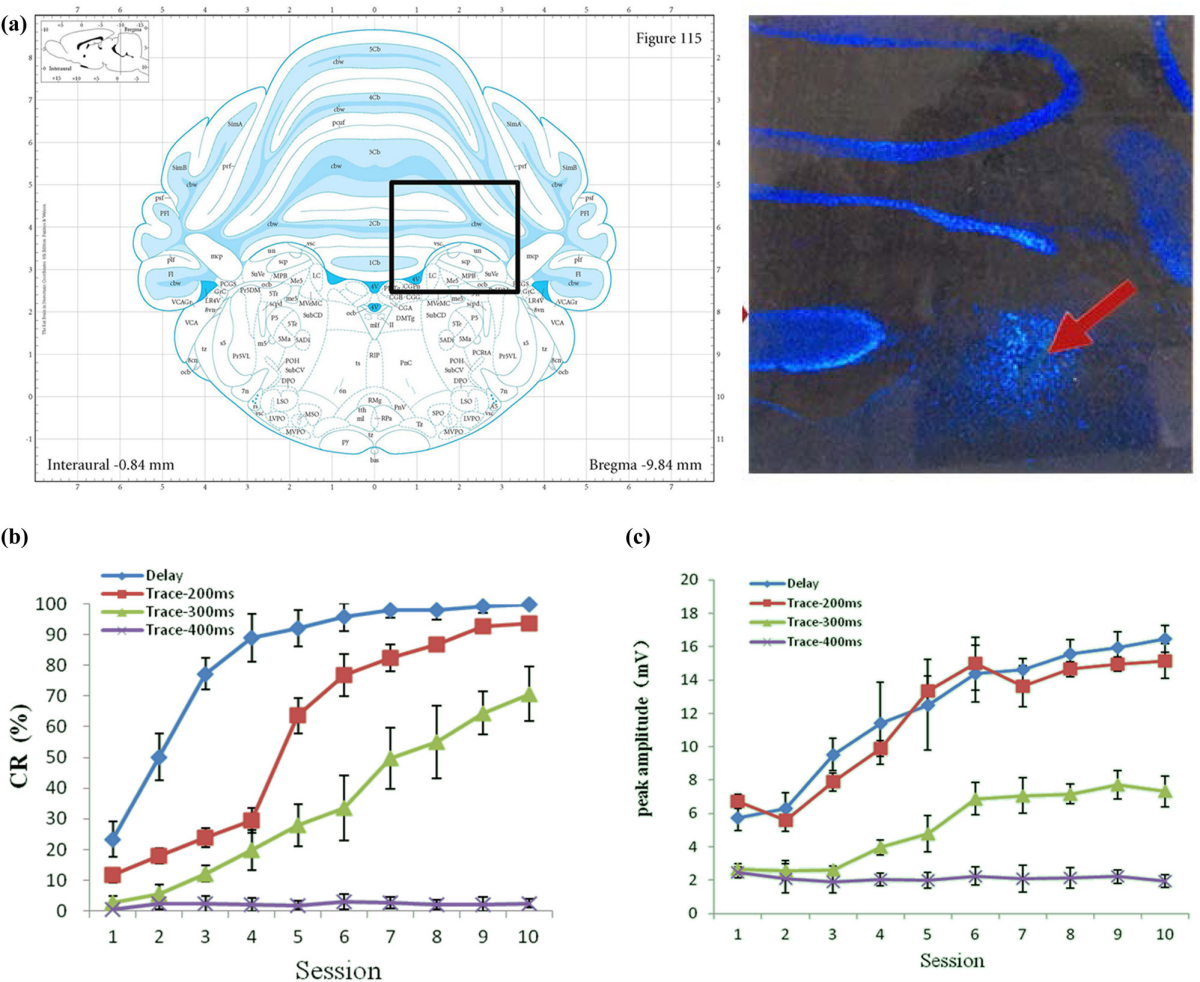
The location of implanted peduncle cerebellar medius-stimulating electrode and the variation pattern of CR-AR and the magnitude of the difference between the maximum active eyeblinks in trace eyeblink conditioning guinea pigs. (a) The red arrow points to the injured brain tissue after electrode implantation (magnification: 100×). (b and c) The line graph of CR-AR and the magnitude of the difference between the maximum active eyeblinks in different groups when constructing eyeblink conditioning models.

After behavior training, the site of electrode implantation was observed on slices. Based on our observation, a total of 28 guinea pigs had correct implantation at the left middle cerebellar peduncle (Figure 2a), out of which six belonged to the Delay group, five to the Trace-200ms group, six to the Trace-300ms group, five to the Trace-400ms group, and six to the control group. The two guinea pigs with incorrect electrode implantation were excluded from the statistical analysis. None of the animals had infections.

After 10 consecutive days of behavior training, the Delay, Trace-200ms, and Trace-300ms groups showed conditioned stimulus-associated active eyeblinks within 200 ms before unconditioned stimulus onset. On the 10th day of training, the CR-acquisition rate (AR) of all three groups was greater than 60%, indicating that the eyeblink conditioning model was established successfully. Furthermore, the CR-AR increased gradually with time. In the Trace-400ms group, however, the CR-AR on the 10th day of 2.50 ± 1.37% was not significantly different from that on the 1st day. Likewise, the control guinea pigs had a CR-AR of 1.66 ± 1.03% on the 10th day of training, which was not significantly different from that on the 1st day. Since there were no conditioned stimulus-associated active eyeblinks within 200 ms before unconditioned stimulus onset, the eyeblink conditioning model was, therefore, not established (Figure 2b). During the 10-day training, the magnitude of the difference between the maximum active eyeblinks of the Delay, Trace-200ms, and Trace-300ms groups increased gradually with time, and their values on the 10th day were significantly different from those on the 1st day. Nevertheless, the magnitude of the difference between the maximum active eyeblinks of the Trace-400ms group showed no significant difference between the 10th and 1st days, which again indicated that eyeblink conditioning was not established in these animals (Figure 2c). These results suggest that a microcurrent pulse could substitute the traditional sound and light as the conditioned stimulus to stimulate the middle cerebellar peduncle directly. In addition, using corneal blowing as the unconditioned stimulus, two types of conditioned reflex, namely delay eyeblink conditioning and trace eyeblink conditioning, could be established successfully with certain time intervals. It indicates that for time-associated events, the cerebellar cortex could independently complete the process of associative learning and memory.

According to our results, using specifically activated lateral pontine nucleus neurons and the projected middle cerebellar peduncle fibrous bundles as the conditioned stimulus, as well as an air puff to the left eyelid as the unconditioned stimulus, the trace eyeblink conditioning model with a conditioned stimulus-unconditioned stimulus time interval of 200 ms could be established successfully.

## 4 Discussion

As suggested by previous studies, during eyeblink conditioning, the cerebellar cortex receives signals from the mossy fiber and climbing fiber that are, respectively, activated by conditioned stimulus and unconditioned stimulus, and then, the signals are gathered and processed in the cerebellar cortex and deep nuclei, followed by appropriate active eyeblinks within 200 ms before unconditioned stimulus onset activity initiated by downstream motor circuits under the control of contralateral nucleus ruber and other nerves [29,51]. The delayed eyeblink conditioning only requires the participation of the cerebellum-brain stem circuit; therefore, the cerebellum could be directly excited without the involvement of the forebrain structures [52,53,54]. Studies of electrophysiology [29] and functional imaging [21,22] have also demonstrated that during delayed eyeblink conditioning, the cerebellar cortex is significantly activated in areas specific to motion and memory. Nevertheless, a study has suggested that in patients with cerebellar degeneration, eyeblink conditioning with a time interval of 1,000 ms could still be achieved [4]. Therefore, whether the cerebellar cortex can independently exert the function of temporal information processing is still controversial. In contrast to the models above, we used a microcurrent pulse to excite the input mossy fiber of the cerebellar cortex directly. It avoided the traditionally used forebrain–cerebellum circuit [2]. Gao et al. [55] showed that the cerebellar excitatory nucleocortical closed-loop circuitry relays premotor signals in a corollary discharge fashion. Therefore, our design excluded the influence of external factors as much as possible and facilitated the analysis of the cerebellum in terms of its independent role in associative learning and memory.

The presence of a time interval in the trace eyeblink conditioning model increased the difficulty of the cerebellar cortex to extract and process information based on the association between the conditioned and unconditioned stimuli. With unchanged conditioned stimulus and unconditioned stimulus, the establishment of the Trace model is harder than that of the Delay model; nevertheless, with an increasing time interval, the Trace model could be achieved with a lower CR-AR [56]. In this study, the Trace-200ms and Trace-300ms groups did not reach a CR-AR greater than 60% until the middle (6th day, 76.80 ± 6.76%) and late (9th day, 64.5 ± 7.00%) phases, but they still acquired trace eyeblink conditioning successfully. It indicates that when the time of occurrence of the conditioned and unconditioned stimuli does not overlap, the cerebellar cortex still establishes trace eyeblink conditioning independently, as suggested by a previous study [30]. The Albus–Marr Calculation Model suggests that when a conditioned stimulus-excited mossy fiber discharge does not overlap with the unconditioned stimulus-driven climbing fiber discharge, the ability of the cerebellum to establish trace eyeblink conditioning would be weakened in comparison to the condition of the overlapped conditioned and unconditioned stimuli. For trace eyeblink conditioning with a time interval ≥400 ms, additional input of nervous signal is needed to maintain the persistent excitation of mossy fiber, and thus, the time interval between climbing fiber could be compensated [57]. When the time interval was set at 400 ms, microcurrent pulse stimulation of the middle cerebellar peduncle failed to achieve trace eyeblink conditioning, and this is supported by the literature [30,55,56], as well as by Thompson and Steinmetz [2], Lee et al. [58], and Li et al. [59], who suggested that the cerebellum plays a role in the timing of associative learning and memory.

On the other hand, the studies by Kalmbach et al. [60,61,62] showed that when establishing a Delay/Trace double eyeblink conditioning, the achievement of trace eyeblink conditioning under a time interval >400 ms needs multistage forebrain impulse to mediate the process of learning and memorizing. Another study showed that patients with cerebellar degeneration could acquire trace eyeblink conditioning with a stimulation interval of 400 ms [4]. These results are different from our study’s, and some reasons might account for the differences. First, the animals used are rodents, whereas the above study used rabbits and even humans as their study subjects. Though the basic components of the cerebellar structures are similar, we cannot deny that the volume of the brain and the complexity of the brain structures among different species might cause some differences in the results [63,64]. Cats [65], rabbits [66], and mice [67] show conditioned responses at 400 ms. Our results will be validated in rabbits in future experiments. Second, Kalmbach et al. [60,61,62] used electric stimulation to the medial prefrontal cortex as the conditioned stimulus, and the excitation was conducted to the cerebellar cortex via the lateral pontine nucleus, whereas in our study, we directly stimulated the mossy fiber connected to the cerebellar cortex. Besides, the location of the microcurrent pulse excitation and the intensity of the microcurrent were also different. The intensity of the electric stimulation was only 20–80 μA, which is far lower than that used by Kalmbach et al. (200 mA). Indeed, large currents might cause peripheral sensory stimulation, which further triggers forebrain stimulation induced by other projection neurons and participate in the process of trace eyeblink conditioning as a compensatory signal.

Unfortunately, the present study was not designed to examine what occurs at the cellular level. Nevertheless, it is known that in cerebellar learning, the instruction signals for long-term depression (LTD) are from the climbing fiber along with the parallel fiber input [68]. The LTD plays a role in the output signal through the disinhibition of cerebellar nucleus neurons that receive GABAergic signals from Purkinje cells. A climbing fiber stimulus will elicit a voltage and calcium signal in Purkinje cells, promoting the induction of LTD [68]. Based on this model, we will analyze the number and morphology of dendritic spines in cerebellar Purkinje cells and granular cells and will also observe whether there is differential expression of mRNA, miRNA, lncRNA, and related proteins, thereby exploring possible downstream molecular targets. Ammann et al. [69] described that the motor cortex lead (in advance to the cerebellum) the generation of conditioned responses. Therefore, we will test in the future whether the electrical stimulation of the middle peduncle can backward activate the motor cortex circuits.

In conclusion, a microcurrent pulse stimulation was used to excite the lateral pontine nucleus-middle cerebellar peduncle circuit, thereby avoiding interference factors from either the external environment or other brain areas. It could be a good model for studying the function and molecular mechanism of the cerebellum during the establishment of eyeblink conditioning.
